# Efficacy of oral fluralaner (Bravecto) against *Tunga penetrans* in dogs: A negative control, randomized field study in an endemic community in Brazil

**DOI:** 10.1371/journal.pntd.0010251

**Published:** 2022-03-14

**Authors:** Katharine Costa dos Santos, Rafael Marin Chiummo, Anja Regina Heckeroth, Eva Zschiesche, Paula Elisa Brandão Guedes, Tatiani Vitor Harvey, Anderson Vieira de Jesus, Anaiá da Paixão Sevá, Joana Thaisa Santos de Oliveira, Zelina dos Santos Freire, Jürgen Krücken, Fernando de Almeida Borges, Georg von Samson-Himmelstjerna, Renata Santiago Alberto Carlos

**Affiliations:** 1 UESC, State University of Santa Cruz, Department of Agricultural and Environmental Sciences, Postgraduate Program in Animal Science, Ilhéus, Bahia, Brazil; 2 MSD, Animal Health Innovation GmbH, Schwabenheim, Germany; 3 UFMS, Federal University of Mato Grosso do Sul, Faculty of Veterinary Medicine and Animal Science, Campo Grande, Mato Grosso do Sul, Brazil; 4 Freie Universität Berlin, Institute for Parasitology and Tropical Veterinary Medicine, Berlin, Germany; 5 UESC, State University of Santa Cruz, Department of Agricultural and Environmental Sciences, Postgraduate Program in Animal Science, Ilhéus, Bahia, Brazil. CNPq Reseracher - PQ2; QIMR Berghofer Medical Research Institute, AUSTRALIA

## Abstract

The sand flea *Tunga penetrans* is one of the zoonotic agents of tungiasis, a parasitic skin disease of humans and animals. The dog is one of its main reservoirs. This negatively controlled, randomized, double-masked clinical trial evaluated the therapeutic and residual efficacy of fluralaner for treatment of dogs naturally infested with *T*. *penetrans*. Sixty-two dogs from an endemically affected community in Brazil were randomly assigned to either receive oral fluralaner (Bravecto chewable tablets) at a dose of 25 to 56 mg fluralaner/kg body weight, or no treatment (31 dogs per group). Dogs were clinically examined using a severity score for acute canine tungiasis (SCADT), parasitological examinations as defined by the Fortaleza classification, and pictures of lesions on days 0 (inclusion and treatment), 7 ± 2, 14 ± 2, 21 ± 2, 28 ± 2, 60 ± 7, 90 ± 7, 120 ± 7 and 150 ± 7. The percentage of parasite-free dogs after treatment was >90% between days 14 and 90 post-treatment with 100% efficacy on study days 21, 28 and 60. Sand flea counts on fluralaner treated dogs were significantly lower (p<0.025) than control dogs on all counts from day 7 to 120. The number of live sand fleas on treated dogs was reduced by > 90% on day 7, > 95% on days 14 and 90, and 100% from day 21 to 60, and with a significant difference between groups from day 7 to 120. From day 7 to day 120, mean SCADT scores were significantly reduced in treated dogs with a mean of 0.10 compared to 1.54 on day 120 in untreated dogs. Therefore, a single oral fluralaner administration is effective for treating and achieving long lasting (> 12 weeks) prevention for tungiasis in dogs.

## Author summary

Tungiasis is a dermatological disease that affects humans and animals infested with sand fleas (*Tunga* spp.), especially in communities exposed to contaminated sandy and dry soil, where living conditions are precarious and access to basic health is limited. These sand fleas penetrate host skin and cause severe acute and chronic injuries that can dramatically compromise the host’s health. Dogs are a primary reservoir and disseminator of these fleas in South America. In this clinical field trial, the efficacy of a single oral dose of fluralaner (Bravecto chewable tablets) was evaluated for treatment and prevention of tungiasis in dogs in an endemic area of Brazil. The treatment was assessed through absence of live sand fleas and resolution of characteristic tungiasis skin lesions. Fluralaner was 100% effective from the third to the eighth week post-treatment, eliminated live sand fleas and protected against new infestations during the study. Therefore, fluralaner proved to be an excellent therapeutic option for treatment and a highly effective control option for canine tungiasis.

## Introduction

Tungiasis is a parasitic skin disease caused by sand fleas of the genus *Tunga*, highly neglected in endemic socioeconomically vulnerable communities, where it affects a wide range of hosts, especially humans, dogs, and pigs but less often also cattle, goats as well as other mammals such as rats. Apparently, dogs are the main animal reservoirs of the zoonotic species *T*. *penetrans* in South America [1,2]. The disease mainly occurs in Latin America, the Caribbean and sub-Saharan Africa including Madagascar [3], with high prevalence rates in human and animal populations from slums, peripheral urban communities, fishing villages, and rural and indigenous communities, where it becomes a major public health problem [4,5].

Maintenance of tungiasis in endemic areas may occur mainly due to the lack of control and inadequate handling of *T*. *penetrans* reservoirs, including dogs, cats, pigs, and rodents, which facilitates spread of eggs of this parasite in the environment. In South America, dogs play an extremely relevant role in the disease transmission cycle, and their frequent contact with people e.g. resting in households makes them an important risk factor for human infestation [6,7].

Adult females *T*. *penetrans* penetrate into skin over a period of minutes to a few hours, where the fleas mature and then release their eggs to the environment. After approximately 4 to 6 weeks the fleas die *in situ* [8,9]. Before dying, the flea infestation causes acute and chronic injuries that trigger clinical signs including local inflammation, pain and itching [10,11]. High parasitic loads can cause serious injuries resulting from self-trauma, deformities, loss of digits, secondary bacterial infections, and septicemia [12]. Consequently, tungiasis dramatically decreases the quality of life for infested individuals [13] and represents a major global One Health issue [14]. Clinical signs and resulting consequences are similar for infested dogs and people [6]. In dogs, a small number of fleas can lead to considerable morbidity presenting as local pain, ulcers, necrosis, hyperemia and edema [10,15].

Reported tungiasis prevalence in Brazilian dogs ranges from 0.7% to 83.9% [13,16]. These results indicate the important role that dogs play in the disease transmission cycle in endemic and non-endemic areas because of their frequent contact with people and other animals, and because they spread the parasite in the environment [17]. In some Brazilian communities, the incidence of this disease can be constant throughout the year, which makes control particularly difficult in the affected communities [18]. In other areas there is an infestation peak in the dry season [19].

Fluralaner is a potent acaricide and insecticide from the family of isoxazolines, a class of systemic antiparasitic agents, which have high and long-term efficacy and can provide protection against ectoparasites [20,21]. This isoxazoline is approved in different formulations in more than 80 countries, including Brazil, for the treatment and control of flea and tick infestations of dogs and cats, and for the treatment of poultry mite infestations. In many countries, fluralaner is also approved for the treatment of demodectic and sarcoptic mange and feline ear mite infestations. Following oral administration to dogs at 25 mg/kg of body weight, fluralaner has a half-life of 12 to 14 days [22]. Fluralaner (Bravecto chewable tablets, MSD Animal Heath) is a systemically administered insecticide that delivers long lasting efficacy against ectoparasites infesting dogs [23]. Therefore, this drug could provide an effective option for treatment and prevention of canine tungiasis. Currently, there are no treatments commercially available with proven and/or long lasting efficacy against *T*. *penetrans* in dogs. Effective control of tungiasis in dogs indirectly contributes to the reduction of infestations in humans [17]. This clinical trial aimed to verify the tungicidal efficacy of a single oral dose of fluralaner (Bravecto chewable tablets) in dogs naturally infested with, and facing ongoing exposure to, *T*. *penetrans*.

## Material and methods

### Ethics statement

The study was approved by the Ethics Committee for Animal Experimentation (CEUA) of the State University of Santa Cruz (UESC), Ilhéus, Bahia, Brazil, under protocol number 020/2019. The study was also conducted considering the principles of “Good Clinical Practice” VICH GL9 (GCP) adopted by the Committee for Medicinal Products for Veterinary Use (CVMP), European Medicines Agency (EMA), in June 2000 (CVMP / VICH / 595/98-Final) [24].

### Study area and population

The study was conducted in the semi-rural community Vila Juerana, located in the city of Ilhéus, in the State of Bahia [13] from November 2019 to April 2020. The community is located at a tourist region of Costa do Cacau, with south latitude 14° 47’ 00” and west longitude 39° 03’ 00”, about 640 m from the Atlantic Ocean and it is endemic for tungiasis. Average annual temperatures in the municipality vary between 22 and 25°C [25] and there is abundant rainfall distributed throughout the year. This region was previously described as having a high prevalence of tungiasis in dogs during throughout the year [5].

The study population consisted of 62 dogs with active tungiasis, characterized by the presence of at least one live sand flea skin lesion classified as stage II or III by Fortaleza classification [26]. After clinical evaluation, for inclusion, dogs with no clinical signs other than tungiasis were enrolled in the study, regardless of sex, age, and breed. As exclusion criteria, dogs treated with fluralaner less than 90 days prior to day 0 or with other shorter acting ectoparasiticides within 14 days prior to day 0 (isoxazolines, amitraz, fipronil, macrocyclic lactones or pyrethroids) were not allowed and also pregnant female dogs were not included in the study.

Owners were asked to sign an informed consent before study initiation. Study dogs remained in the care of their owners and with their usual routines and level of restriction. The dogs were allowed to roam freely in the community. Dogs from different groups were in the same environment and were able to encounter one another during the study.

### Study design

This field study was randomized, negatively controlled, and double-masked. The study area was selected due to the availability of animals, previously found by Harvey and collaborators [13] as a place of high prevalence of the parasite in dogs.

27 dogs in each study group were determined as sufficient to demonstrate superiority of the Treatment Group (TG) in comparison to Control Group (CG) with estimated efficacy rates (“parasite free animals”) of 95% in the TG and 60% in the untreated CG, when the (one-sided) level of significance is set to α = 0.025, with a power of 1-β = 0.8. Assuming a drop-out rate of 10%, approximately 30 dogs per study group were targeted to be enrolled into the study in a 1:1 ratio.

The dogs were recruited through earlier knowledge of the owners due to the prior epidemiological survey carried out at the village. The general study design is outlined in a CONSORT flow chart [27] in S1 Fig. On day 0, 62 dogs were randomly distributed (using a prior computer generated list) to one of two experimental groups (31 dogs each) and implanted with a numerically coded microchip for individual identification. The clinical history of each dog was recorded; each dog received a complete physical examination and the skin examined for parasites.

The primary outcome measure was the number of dogs without embedded female *T*. *penetrans*. Secondary outcome measures were the number of fleas per dog and the severity of the disease quantified by a severity score for acute dog tungiasis (SCADT).

Study activities were distributed between two teams of investigators. One team assigned the animals to groups and administered the treatment and did not participate in clinical evaluations. The other team completed the clinical evaluations and inspections of the dogs, and all members of this team were masked to treatment assignments. Treatments were administered in accordance with label directions, and the dogs in the Treatment Group (TG) received a single oral fluralaner dose (Bravecto chewable tablet, MSD Animal Health) at the approved dose rate of 25–56 mg fluralaner/kg body weight on day 0. Dogs in the CG remained untreated. After administration, all study animals received the same moist commercial dog food and owners were not informed to which group their dogs were assigned. During the study, dogs were treated for any observed secondary complications of tungiasis and for any other routine health needs. Administration of any other parasiticidal drug or product with insecticidal activity was prohibited.

Dogs were evaluated weekly in the first month after treatment and monthly thereafter with 9 scheduled examinations for each dog including days 0 (enrolment and treatment), 7 ± 2, 14 ± 2, 21 ± 2, 28 ± 2, 60 ± 7, 90 ± 7, 120 ± 7, and 150 ± 7.

### Clinical evaluation, skin inspection and lesion documentation

At each evaluation visit, the dogs received a general physical examination and detailed skin inspection made by separating hair over the body to search for tungiasis lesions. Paws, limbs, tail, mammary glands, abdomen, testicles, and nose were examined. Before examination, the dogs’ paws were cleaned using a brush and water to improve detection of all lesion stages. Identified lesions were staged according to the Fortaleza Classification [26].

A SCADT score was assigned to each dog to record its clinical status throughout the study. Each clinical sign was scored for each affected area, and the results added to obtain the SCADT (Table 1). The maximum possible score on this classification is 27. If an animal exceeded 22 in the SCADT score, the dog would be treated by surgical removal of *Tunga* spp. neosomes and excluded from the study for ethical reasons; however, this procedure was not needed during the trial. All lesions were classified, quantified, photographed and documented in a parasitological skin exam record.

**Table 1:** Severity score for acute dog tungiasis (SCADT) used at each clinical exam of dogs in a trial of the efficacy of oral fluralaner for treatment of this parasite.

Characteristic clinical signs	Number of topographic sites affected	Score assigned
**Hyperemia and/or edema** ^**a**^	1–5	1
** **	6–10	2
** **	11–16	3
**Pain on digital pressure**	1–5	1
** **	6–10	2
** **	11–16	3
**Suppuration and/or abscess formation** ^**a**^	1–5	1
** **	6–10	2
** **	11–16	3
**Clustering of lesions** ^**b**^	1–5	1
** **	6–10	2
** **	11–16	3
**Fissure (s)** ^**a**^	1–5	1
** **	6–10	2
** **	11–16	3
**Skin ulceration** ^**a**^	1–5	1
** **	6–10	2
** **	11–16	3
**Mutilation** ^c^		2
**Altered gait/lameness**		3
**Ectopy of lesions**		0.5 ^d^

^**a**^ Irrespective of number of foci and size of the area involved on a designated topographical site.

^**b**^ Three or more lesions in close proximity (1–2 mm apart).

^**c**^ Mutilation of lesions reflects intense itching.

^**d**^ For each ectopic discrete body part involved up to a maximum of eight ectopic sites; maximum 4 points.

Therefore, the maximum score (SCADT) for an individual dog will be 27 (23+4).

### Statistical analysis

Statistical analysis to assess treatment efficacy of Bravecto was performed using the SAS software package (SAS Institute Inc., Cary, NC, USA, version 9.4), with the dog as the experimental unit. Primary efficacy was based on the percentage of parasite-free dogs (dogs free from live sand fleas, stages II and III). The 95% confidence limits for percentage of dogs without living *T*. *penetrans* were calculated as Wilson score intervals. At each post-treatment evaluation time point, Fisher’s Exact Test (one-sided, using the continuity correction of Casagrande, Pike and Smith [28]) was used with a significance level of α = 0.025 to compare the percentage of parasite-free cases between the CG and the TG. Resulting p-values, Odd’s ratios (OR) and 95% confidence limits for OR were obtained from PROC FREQ using the Taylor series approach.

Secondary efficacy (SE) evaluations were based on the number of live sand fleas (stages II and III) and SCADT scores. At each post-treatment evaluation time point, treatment effectiveness was calculated using Abbott’s formula as follows:

$$\mathrm{Efficacy}\;[\%]=\frac{{\bar{x}}_{C}-{\bar{x}}_{T}}{{\bar{x}}_{C}}∙100$$

where            ${\bar{x}}_{C}$            is the geometric mean of live flea counts in the CG;

                                    ${\bar{x}}_{T}$ is the geometric mean of live flea counts in the TG.

To allow the calculation in case of zero count, the geometric mean was calculated as follows:

$$x_{g}={\left(\prod_{i=1}^{n}\left(x_{i}+1\right)\right)}^{\frac{1}{n}}‐1$$

To confirm the results of secondary efficacy results for each time point, mean live flea counts in the TG and CG were log-transformed (x’ = log_e_ (x + 1)) and compared using a two-sided two-sample t-test with the level of significance set to α = 0.05. Cohen’s d as the effect size for treatment was determined manually using t-statistic (t) and degrees of freedom (DF) as obtained from PROC TTEST: $d=\frac{2∙t}{\sqrt{\mathrm{DF}}}$.

Treatment effectiveness based on arithmetic means of live flea counts was calculated for additional information and 95% confidence intervals for efficacy [%] were computed where possible.

For an additional comparison of the course of flea counts with time, a repeated measures analysis of variance model was applied to the log-transformed flea counts (PROC MIXED). In this model, study day was the within-subject effect, study group and group-day interaction were the between-subject effects.

An additional evaluation was done in the CG sand flea counts. Two-sided t-tests for paired observations were used to estimate the difference of flea counts between day 0 and each time point after day 0.

A generalized linear model with log flea counts as dependent variable and a fixed effect, Day, for independent variable was used to estimate the difference between the day 0 flea count and the counts after treatment for the CG with the level of significance set to α = 0.05.

Mean SCADT scores in the two groups were compared at each post-treatment evaluation time point using Wilcoxon’s Rank Sum Test (exact), with the level of significance set to α = 0.05 (two-sided).

## Results

Study dogs, aged between 4 months and 14 years, were all mixed breed, intact, and weighing between 2.2 and 31.3 kg. On day 0 before treatment, *T*. *penetrans* mean live flea counts were 27.3 ± 36.8 in treated dogs and 14.6 ± 16.6 in untreated dogs, while the flea count per dog was 1–180 fleas on treated dogs and 1–63 on control dogs. Study groups were comparable on Day 0 with regards to the distribution of age, weight, sex and total severity score (SCADT) (Table 2). The flea count in the TG was slightly higher than in the CG.

All dogs that participated in the study were included in the statistical analysis.

**Table 2:** Distribution of age, weight, sex, flea count and total severity score (SCADT) on Day 0.

		Fluralaner treated	Untreated
**Number of dogs [N]**		31	31
**Age [years]** **(mean ± SD, [age range])**		4.3 ± 3.6 [4 months; 14 years]	3.7 ± 2.7 [4 months; 11 years]
**Body weight [kg]** **(mean ± SD, [weight range])**		10.8 ± 6.0 [2.2; 25.6]	10.8 ± 6.5 [2.9; 31.3]
**Sex**	**Female intact**	16 (51.6%)	12 (38.7%)
	**Male intact**	15 (48.4%)	19 (61.3%)
**Flea count** **(mean ± SD, [range])**		27.3 ± 36.8 [1; 180]	14.6 ± 16.6 [1; 63]
**SCADT** **(mean ± SD, [range])**		7.1 ± 4.6 [0; 15.0]	5.4 ± 3.7 [0; 12.5]

Three dogs died during the study: a dog fight (untreated dog), unknown reason (untreated dog), and car accident (treated dog). One untreated dog was removed at the owner’s request. However, results collected before loss of these dogs to the study were valid and were used for statistical analyses. No adverse treatment-related events were observed in any dog in the TG.

### Parasite free results

On day 7, 17/31 treated dogs (54.8%) were flea free and on day 14, 30/31 (96.7%) dogs were flea free. On days 21, 28 and 60 post-treatment, all treated dogs were flea free and efficacy was 100% against *T*. *penetrans*, with treatment also preventing reinfestation. On day 90, 29/31 (93.6%) treated dogs were flea free and without signs of active sand fleas. On day 120, 24/31 (77.4%) and on day 150, 11/30 (36.7%) treated dogs had no active sand fleas. The number of untreated dogs without fleas was significantly lower than treated dogs at all time points post treatment, except on day 150 (Table 3).

**Table 3:** *Tunga penetrans* free dogs at various time points following either a single oral dose of fluralaner or no treatment.

Day	Fluralaner treated		Untreated		p value^b^	OR^c^ [95% CL]
	Flea free (total)	% (95% CL^a^)	Flea free (total)	% (95% CL^a^)		
7	17 (31)	54.8 (37.8–70.8)	5 (31)	16.1 (7.1–32.6)	0.0015	6.31 [1.92; 20.76]
14	30 (31)	96.7 (83.8–99.4)	6 (31)	19.4 (9.2–36.3)	<0.0001	125.00 [14.09; 1108.58]
21	31 (31)	100 (89.0–100.0)	6 (31)	19.4 (9.2–36.3)	<0.0001	not computable
28	31 (31)	100 (89.0–100.0)	6 (31)	19.4 (9.2–36.3)	<0.0001	not computable
60	31 (31)	100 (89.0–100.0)	11 (30)	36.7 (21.9–54.5)	<0.0001	not computable
90	29 (31)	93.6 (79.3–98.2)	13 (29)	44.8 (28.4–62.5)	<0.0001	17.85 [3.57; 89.19]
120	24 (31)	77.4 (60.2–88.6)	10 (28)	35.7 (20.7–54.2)	0.0013	6.17 [1.96; 19.35]
150	11 (30)	36.7 (21.9–54.5)	4 (28)	14.3 (5.7–31.5)	0.0488	3.47 [0.95; 12.66]

^**a**^ CL: Confidence Limits

^**b**^ Fisher’s Exact Test, α = 0.025

^**c**^ OR: Odd’s Ratio, CL: Confidence Limits

**Fig 1:**
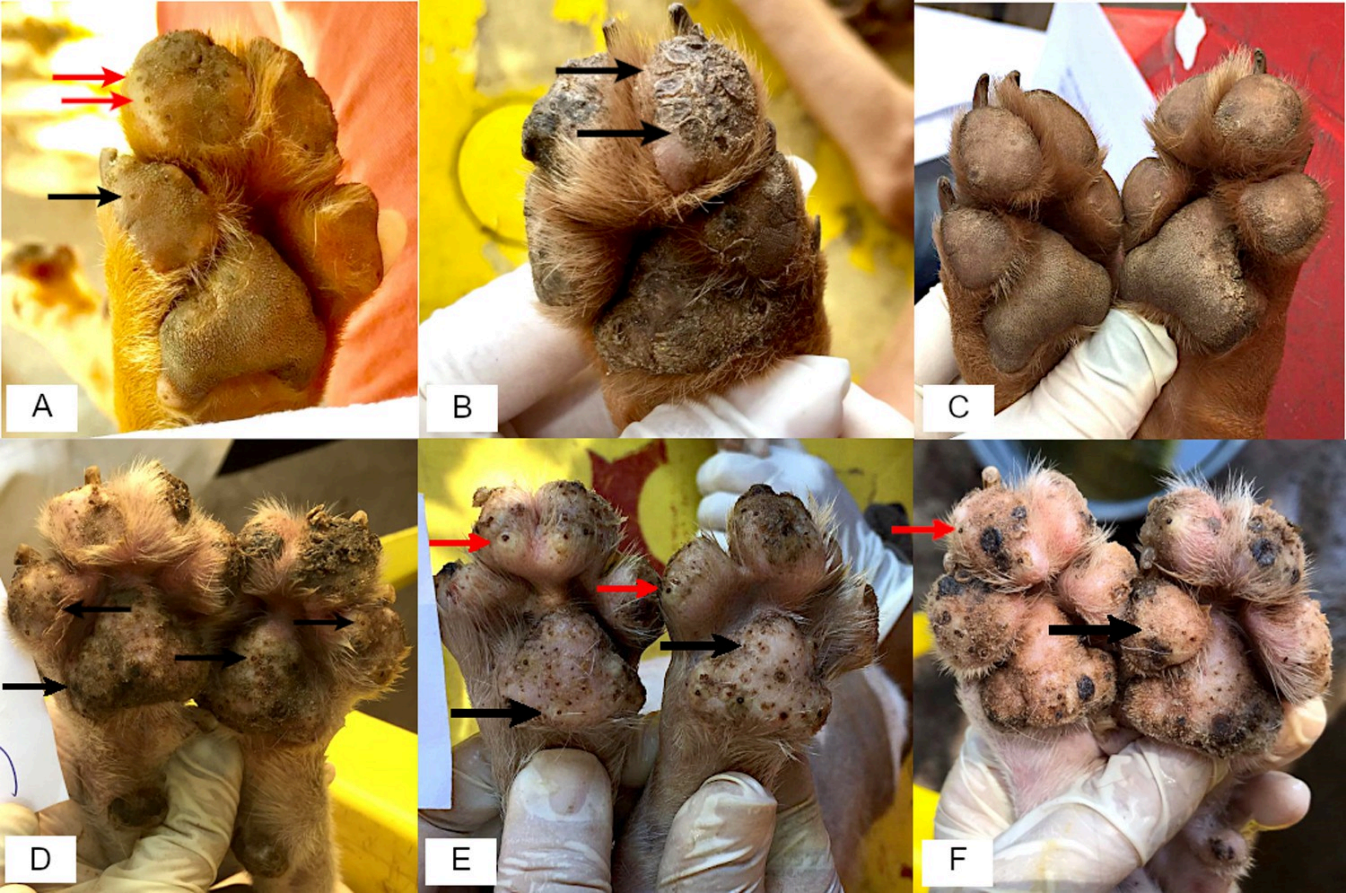
Front paws of fluralaner treated and untreated dogs. (A) Treated dog on day 0 showing multiple stage II (example in black arrow) and III (examples in red arrows) *T*. *penetrans* lesions. (B) Same dog day 7—no active lesions and visible parasite involution scars (stage V–examples in black arrows). (C) Same dog day 21—paw pad epithelium has regrown and pad is flea free (D) Untreated dog, on day 0 with multiple active flea lesions on the paw pads of the forelimbs (examples in black arrows). (E) Same untreated dog on day 7—numerous flea lesions in stages II (examples in black arrows) and III (examples in red arrows). (F) Same untreated dog—day 21, with flea active lesions II (example in black arrow) and III (example red arrow), indicating reinfestation. Notice that there are more lesions in Fig 1A, 1D, 1E and 1F than the arrows are showing. Source: author’s photographic collection.

At day 0, dogs of both groups showed multiple signs of acute tungiasis such as living embeded female fleas in all stages of development, hyperemia, edema, ulcers and hyperkeratosis. Fig 1 shows the elimination of all viable fleas in a treatment group dog after the treatment and the presence of viable fleas in the control group throughout the study, including the occurrence of new infestations. At day 7, viable fleas were not found anymore but there were still many scars visible at the sites where fleas had died in situ. Already at day 21, the foot pads of treated dogs looked almost normal with few signs of chronic tungiasis such as circular impressions at positions where fleas had been embedded. The skin was also still rougher than that of a dog never infested by sand fleas.

### Secondary efficacy and clinical signs

**Fig 2:**
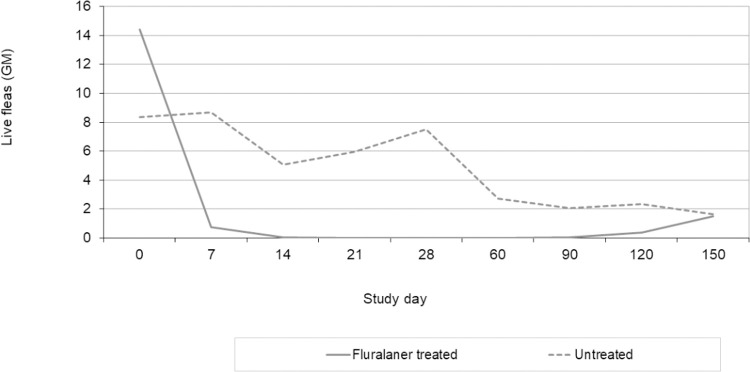
Geometric means of log-transformed live flea counts per study group over time.

Geometric means of live flea counts are shown in Fig 2. Fluralaner (Bravecto chewable tablets) secondary treatment efficacy on day 7 (reduction in total number of live sand fleas) was 91.5%, while from day 14 to 90, efficacy was > 95%, and was 100% from day 21 to 60. On day 120, efficacy was 84.4%. Similar results are obtained for efficacy based on arithmetic means of live flea counts. The geometric mean live sand flea count was significantly lower in treated dogs (p ≤ 0.0036), from days 7 to 120. There was no significant difference between treated and untreated dogs on day 150 with efficacy < 10%. (Table 4). The effect sizes calculated as Cohen’s d values were large for all time points from day 7 to day 120 post treatment with the difference between the means being larger than the pooled standard deviation (Cohen’s d < -1). In fact, the effect size was even very large (d < -1.2) on days 7, 60 and 90 and huge (d < -2) between days 14 and 28 (Table 4) applying the descriptors for Cohen’s d magnitude by Sawilowsky [29].

**Table 4:** Live *Tunga penetrans* counts (secondary efficacy and p values) on fluralaner treated and untreated dogs at various time points following treatment.

Day	Geometric mean live flea counts			Arithmetic mean live flea counts			95% CL of AM efficacy^a^	p-value^b^*	Cohen’s d (Effect size)
	Fluralaner treated	Untreated	Efficacy [%]	Fluralaner treated	Untreated	Efficacy [%]			
0	14.40	8.36	-	27.26	14.58	-	-	-0.0751	0.305
7	0.74	8.67	91.5	1.48	20.10	92.6	[82.39; 98.26]	<0.0001	-1.787
14	0.05	5.09	99.1	0.10	13.06	99.3	[97.01; 100.39]	<0.0001	-2.536
21	0.00	5.95	100	0.00	17.52	100	not computable	<0.0001	-2.691
28	0.00	7.51	100	0.00	22.87	100	not computable	<0.0001	-2.969
60	0.00	2.74	100	0.00	10.83	100	not computable	<0.0001	-1.916
90	0.05	2.07	97.8	0.06	7.45	99.1	[92.16; 100.84]	<0.0001	-1.633
120	0.37	2.37	84.4	0.87	9.32	90.7	[54.54; 99.40]	0.0036	-1.015
150	1.52	1.62	6.3	4.97	2.64	0	[-569.9; 94.88];	0.8687	-0.048

^a^ CL: Confidence Limits, AM: Arithmetic Mean

^b^Two-sided two-sample t-test, α = 0.05

Using a repeated measures analysis of variance model on log-transformed flea counts (Fig 2) where study day is the within-subjects effect and study group and group-day interaction are the between-subjects effect, a significant difference between study groups is shown (p<0.0001). There is also a significant change of flea counts with time (day: p<0.0001) and a significant difference between the course over time in both study group (group-day interaction: p<0.0001).

Table 5 provides the analysis on flea data for the CG. A positive difference denotes an increase with time, a negative difference denotes a decrease with time. The results showed that the flea counts on day 0 were significantly different from those recorded on days 90 and 150 (p-value < 0.05). Most Cohen’s d values for time points compared to the begin of the study were small or medium, only for day 150 a very large effect with substantially decreased flea counts in the control group was observed (Table 5).

**Table 5:** Two-sided t-test for paired observations, estimating the difference of flea counts between day 0 and the time points after treatment for the CG.

Comparison	Mean difference (flea counts)	Standard Error	95% Confidence Limits of the mean	Range [min; max]	p-value	Cohen’s d (Effect size)
day 0 vs day 7	5.516	20.936	[-2.163; 13.196]	[-35; 81]	0.1528	0.537
day 0 vs day 14	-1.516	12.977	[-6.285; 3.251]	[-21; 36]	0.5210	-0.237
day 0 vs day 21	2.935	23.530	[-5.695; 11.566]	[-43; 71]	0.4926	0.252
day 0 vs day 28	8.290	38.981	[-6.008; 22.588]	[-26; 196]	0.2457	0.431
day 0 vs day 60	-2.700	18.447	[-9.588; 4.188]	[-50; 58]	0.4293	-0.297
day 0 vs day 90	-6.517	14.171	[-11.908; -1.127]	[-50; 28]	0.0196	-0.955
day 0 vs day 120	-4.821	19.094	[-12.225; 2.583]	[-49; 44]	0.1927	-0.516
day 0 vs day 150	-11.500	14.180	[-16.998; -6.002]	[-49; 7]	0.0002	-1.651

**Fig 3:**
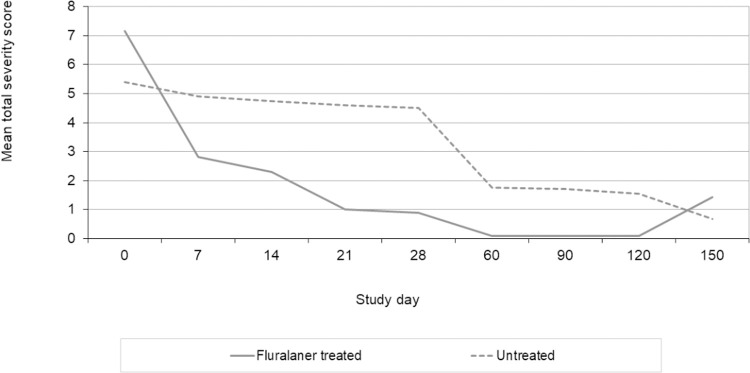
Mean severity score for acute dog tungiasis (SCADT) per study group over time.

Results for SCADT scores are shown in Table 6 and Fig 3.

**Table 6:** Clinical scores as measured by mean SCADT (data by inspection time point by study group and p-values) in fluralaner treated and untreated dogs naturally exposed to *Tunga penetrans*.

Day	Fluralaner treated		Untreated		p-value^a^
	Mean ± SD	Median	Mean ± SD	Median	
0	7.15 ± 4.63	7.00	5.39 ± 3.66	5.00	0.1400^**b**^
7	2.81 ± 2.37	2.00	4.90 ± 3.17	5.00	0.0074
14	2.29 ± 1.74	2.00	4.74 ± 3.20	5.00	0.0017
21	1.00 ± 1.39	0.00	4.61 ± 4.11	4.50	<0.0001
28	0.90 ± 1.60	0.00	4.50 ± 3.60	4.00	<0.0001
60	0.10 ± 0.40	0.00	1.77 ± 2.69	0.00	0.0003
90	0.10 ± 0.54	0.00	1.72 ± 2.46	0.00	<0.0001
120	0.10 ± 0.30	0.00	1.54 ± 2.63	0.00	0.0011
150	1.43 ± 2.56	0.00	0.68 ± 1.29	0.00	0.4701

^**a**^Wilcoxon’s Rank Sum Test (exact), α = 0.05

^**b**^ Wilcoxon’s Rank Sum Test (normal approximation), α = 0.05

**Fig 4:**
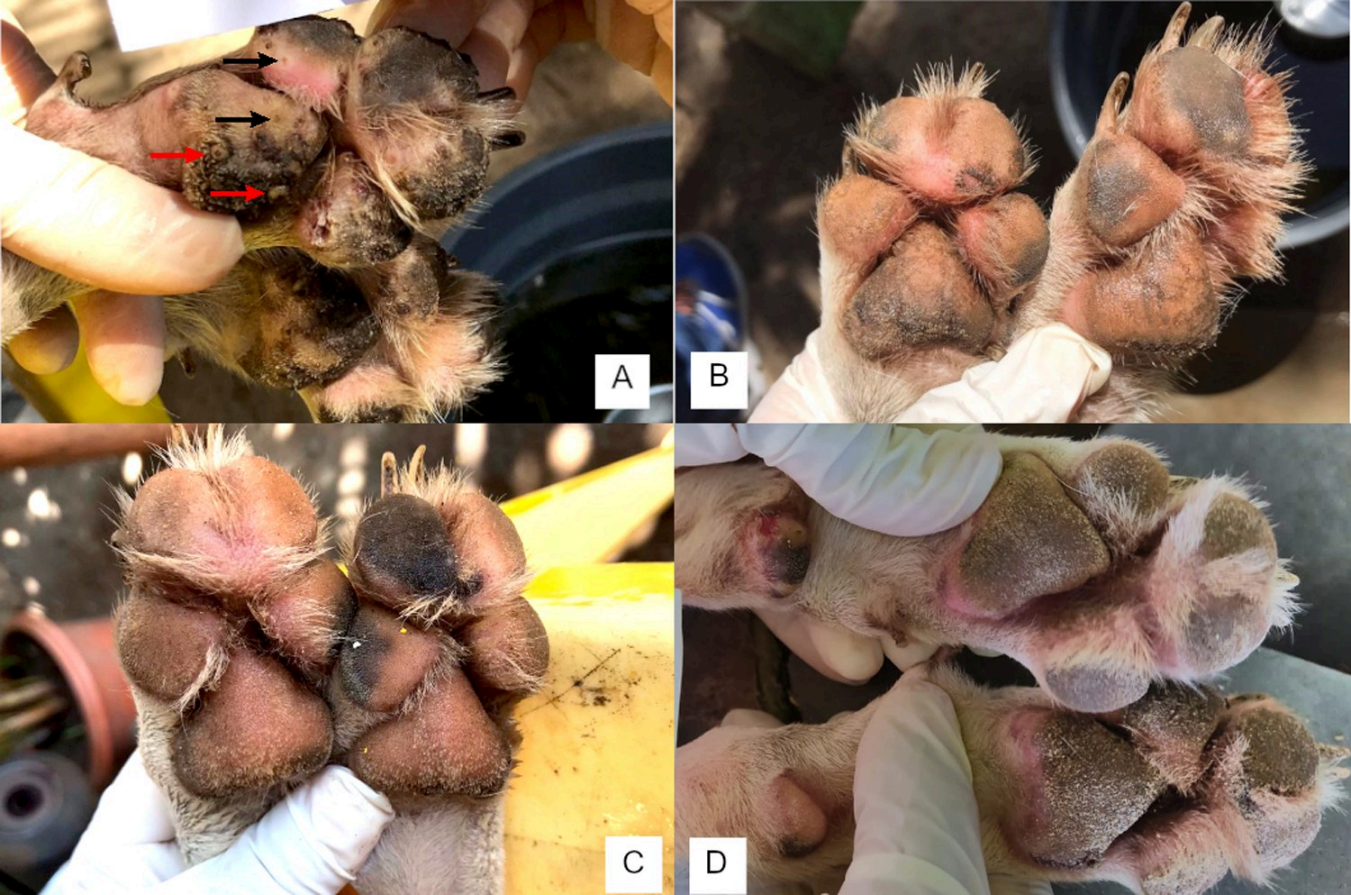
Continued protection and improvement in paw clinical appearance in a fluralaner treated dog. (A) Day 0—multiple stage II (examples marked by black arrows) and III (examples marked by red arrows) lesions caused by *T*. *penetrans*, located on the paw pads. (B) Day 28—no active sand flea lesions. (C) Day 60—paw pad epithelium regrowth and no new infestations. (D) Day 90 –normal paw pads. Notice that there are more lesions in Fig 4A than the arrows are showing.

The mean clinical scores of fluralaner treated dogs improved compared to untreated dogs (Table 6). Following treatment mean total severity scores were significantly lower in treated dogs (p ≤ 0.0074) from day 7 to day 120. Photographs of a treated dog illustrate the sequential improvement after treatment (Fig 4). On day 90, the epithelium of the food pads had completely regenerated (Fig 4D). On day 150 the mean SCADT score was higher in treated dogs, but not significantly different from untreated dogs. Neither at the start nor during the conduct of the study any of the dogs had a SCADT of 22 or above and thus no dogs were excluded from the study due to severe tungiasis.

## Discussion

This study showed that a single oral fluralaner (Bravecto chewable tablet) administration to dogs naturally infested with *T*. *penetrans* provided a high level of efficacy for the primary outcome measure, the percentage of dogs without embedded sand fleas for more than 12 weeks. This high efficacy reached 100% over extended time points. In addition, the treatment was safe as no treatment related adverse events were recorded. Therefore, this treatment could be a new option to treat tungiasis and for the control of this challenging zoonotic parasitosis in the field. To the best of our knowledge, this is the first published clinical study proving the efficacy of fluralaner for treating and preventing *T*. *penetrans* infestation in dogs. For the secondary outcome variables, i.e. the number of fleas per dog and disease severity in terms of the SCADT, efficacy was also very high but there were some differences in the time course. While the kinetic of changes was almost identical between percentage of flea-free dogs and number of fleas, the improvement of the SCADT lagged behind the two other variable. However, even on study day 120 there was only a minimal pathology observed in the treated group. The delayed drop in clinical pathology and also the delayed increase in symptoms after day 120 can be explained by the fact that a lot of the symptoms are caused by local tissue inflammation and only resolve slowly. It is known that even dead fleas in the skin may cause some degree of inflammation before the remnants of the flea have been completely removed by immune and wound healing processes [30].

The systemic distribution of fluralaner may be a key attribute, eliminating the possibility of reducing the effect of the product by rain or bath action, which is a disadvantage of topical treatments [31]. In addition, its indication for a 12-week retreatment interval avoids lack of compliance issues potentially associated with monthly treatment approaches [32].

Adulticidal action of fluralaner against sand fleas was observed at the first examination visit following treatment on day 7 and the onset of activity likely occurs before this point. There is evidence that onset of fluralaner activity against *C*. *felis* in dogs can be observed as soon as 1 hour following treatment [33]. Maximum fluralaner plasma concentrations are reached approximately 24 hours following oral administration to dogs [32,33] and effective concentrations against fleas are reached at even earlier time points. It is possible that the onset of activity against *T*. *penetrans* is not as rapid as against *C*. *felis* because in our study 54.8% of dogs were completely free of live *T*. *penetrans* at the first examination on day 7. Further investigation on the onset of tungicidal activity are required to clarify this difference.

This study found that fluralaner treatment effectively prevented flea reinfestation for at least 120 days during the study period in dogs exposed to potential re-infestation throughout this time. The study was conducted in an area known to have a high risk for re-infestation with favorable characteristics for year-round development and maintenance of *T*. *penetrans* [5,19]. There was evidence of reinfestation in some dogs on day 90 and across the group of treated dogs at day 150 and fluralaner retreatment by day 90 will be required to maintain efficacy.

It is likely that fluralaner treatment effectively interrupts the parasite’s life cycle by decreasing the number of female fleas and reducing the environmental spread of eggs [34,35]. This effect was observed in previous studies showing that fluralaner prevented *C*. *felis* reproduction in households with dogs [21], and reduced egg production by 99.9% within 48 hours after treatment [20]. This impact on parasite life stages in the environment control could be assessed indirectly by the observation of the CG, that from day 60 to 120 had higher percentages of free-flea dogs (>30%), achieving a peak of 44.8% on day 90. The evaluation of the CG sand flea number during the study showed that day 0 was significantly different from days 60, 90, 120, and 150 (p-value < 0.05), supporting this proposal. The owners of the CG dogs confirmed that they did not treat their dogs for tungiasis during the study. This impact of reducing the risk of new infestations could be beneficial for dogs and people living in endemic areas although this needs further evaluation.

In tungiasis, the severity of clinical signs can vary according to the infestation intensity [25]. Nevertheless, it was observed in this study that dogs with sand flea infestation could have a SCADT of zero. On the other hand, it was also seen that occurrence of dermatological lesions associated with other diseases, such as allergic dermatitis, hampered the clinical evaluation. For instance, although there were moments that TG dogs did not have a sand flea infestation, some of them showed acute clinical signs commonly associated with tungiasis. Therefore, these dogs probably had lesions associated with other conditions.

The systematic application of Bravecto chewable tablets has great potential to be of major impact in any One Health directed approach aiming at the improvement of health outcomes for neglected human populations in areas where *T*. *penetrans* is endemic in dogs. It was not possible, due to limitations in the study resources and organization, to also examine the *T*. *penetrans* infection status of the dog owners and local human populations. This is probably the most important limitation of the study. Wherever previous studies have compared prevalence and intensity of infestation with *T*. *penetrans* between humans and companion animals, a strong correlation could be observed [15,36,37]. Relevant animal species were, depending on the local communities, swine, dogs and cats. However, a correlation is no prove of a causal relationship and it is also unclear if the animals are a reservoir for human tungiasis or the other way round or if transmission in both directions are equally important. In a recent case control study by Gitau et al. [38] compared animal keeping habits between *T*. *penetrans* positive and negative persons. However, the study results should be interpreted with considerable care since the case and control groups significantly differed regarding age, sex and income. Indeed, young as well as old, male and low income persons were highly overrepresented in the case group and all these factors are well known to have significant effects on prevalence of tungiasis as for instance shown by Wiese et al. [39]. Moreover, the study found a significantly increased risk of tungiasis due to the presence of chicken although chickens are apparently not infested by *T*. *penetrans* [15] and their presence was found to be protective against severe tungiasis in previous studies [40]. One of the problems of the study by Gitau et al. [38] might be that the authors only conducted univariate analyses and thus several of the variables might be correlated to an unknown degree (such as that it is likely that people who live from subsistence farming are likely to keep several domestic animal species while people being employed might have no domestic animals). Altogether the current data suggest that a village and not an individual person or a household should be the statistical unit for such investigations. Thus, a study design including a number of villages would be needed to determine if there is an impact of control of animal tungiasis on human tungiasis with the village as statistical unit. A good study design would include six villages with control of animal tungiasis and another six villages (matched regarding socioeconomic variables) without any intervention. An optimal design would require another six villages in which also human tungiasis is systematically controlled, e.g. using dimethicone [41]. Since there are no data available regarding the time how long off-host stages of *T*. *penetrans* can survive in the environment, a minimum of one year should be considered for such a longitudinal study. Obviously, this would be a very expensive study which would be far outside of the scope of the current project aiming to identify a reliable tool to prevent canine tungiasis. Despite this limitation, there is a high chance that control of animal tungiasis will be beneficials for humans in the same villages by overall reduction of environmental contamination.

Following the very positive outcomes of this study, e.g. concerning the long-term prevention of *T*. *penetrans* infestation in the treated dogs, the use of Bravecto chewable tablets in a comprehensive One Health based investigation aiming to provide proof-of-principle for the elimination of clinical tungiasis is now urgently needed. Since it has been repeatedly shown in the past that also other animal hosts such as swine or cats may also be involved in the *T*. *penetrans* epidemiology [17,29,42], these animals would require to be included according to the prevailing conditions in such efforts as well. Since human tungiasis is often found in the most under-resourced communities, any application of Bravecto chewable tablets appears only realistic if the product would be donated either by public health authorities and/or philanthropic donors. Such programs are well established in the field of tropical neglected diseases (NTD) where for example billions of treatments have been donated and successfully employed to combat soil-transmitted-helminth, river-blindness or schistosomiasis, to name just a few [43]. In addition to the clinical symptoms further severe negative consequences of tungiasis, including social exclusion due to stigmatization, on infested individuals are especially encountered in the young and old members of affected communities. This certainly calls for serious and sustainable actions to significantly reduce the disability-adjusted life years attributed to this particularly neglected NTD causes in human populations in many parts of the world.

## Conclusion

A single oral administration of Bravecto chewable tablets was highly effective in treating infestation and preventing reinfestation by *T*. *penetrans* on dogs for three months. Treated dogs recovered from the sand flea associated skin lesions and their clinical status was significantly better than untreated dogs. This treatment could play a decisive role in One Health directed tungiasis control approaches in endemic areas where dogs are a primary reservoir for this zoonosis.

## Supporting information

S1 Fig Flow diagram of the progress through the phases of the randomized fluralaner *Tunga penetrans* treatment trial (i.e. enrollment, allocation, follow-up, and data analysis) according to Consolidated Standards of Reporting Trials (CONSORT). (PDF)
