# Supplementary material for: Efficacy of oral fluralaner (Bravecto) against Tunga penetrans in dogs: A negative control, randomized field study in an endemic community in Brazil
Source: PLoS Negl Trop Dis. 2022 Mar 14;16(3):e0010251. doi: 10.1371/journal.pntd.0010251 (PMC8947607; doi:10.1371/journal.pntd.0010251)

Flow diagram of trial progress according to Consolidated Standards of Reporting Trials (CONSORT)

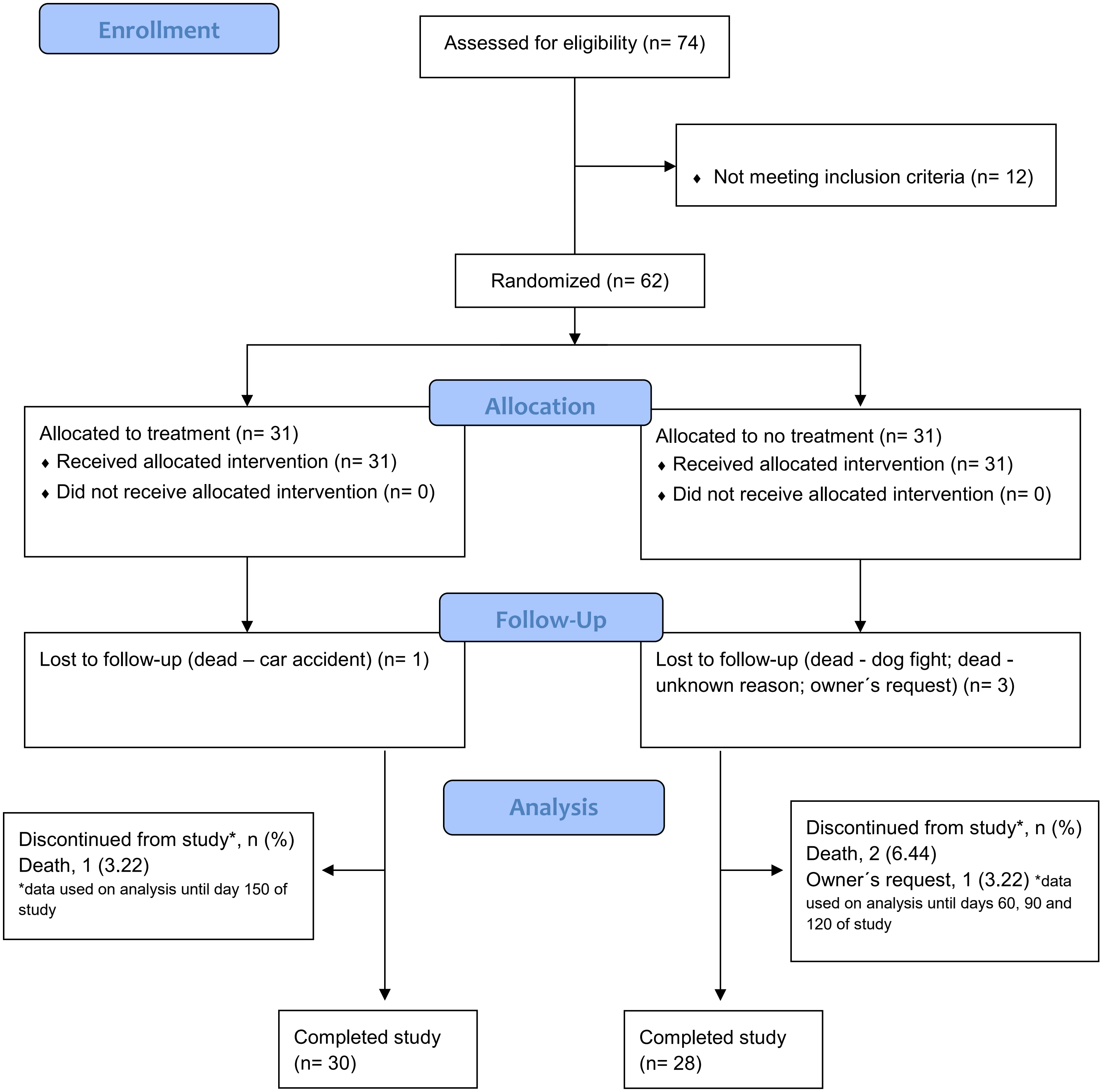

Supplement: S1 Fig — (PDF) [file pntd.0010251.s001.pdf]
